# Gwen Douglas, MB ChB, FRCPsych

**DOI:** 10.1192/bjb.2021.84

**Published:** 2022-04

**Authors:** Peter Shoenberg

**Affiliations:** Email: peter.shoenberg@gmail.com

Formerly Consultant Child Psychiatrist at Sutton Child Guidance Clinic and Clinical Assistant in Psychiatry in the Departments of Obstetrics and Psychotherapy at University College Hospital, London, UK

**Figure:**
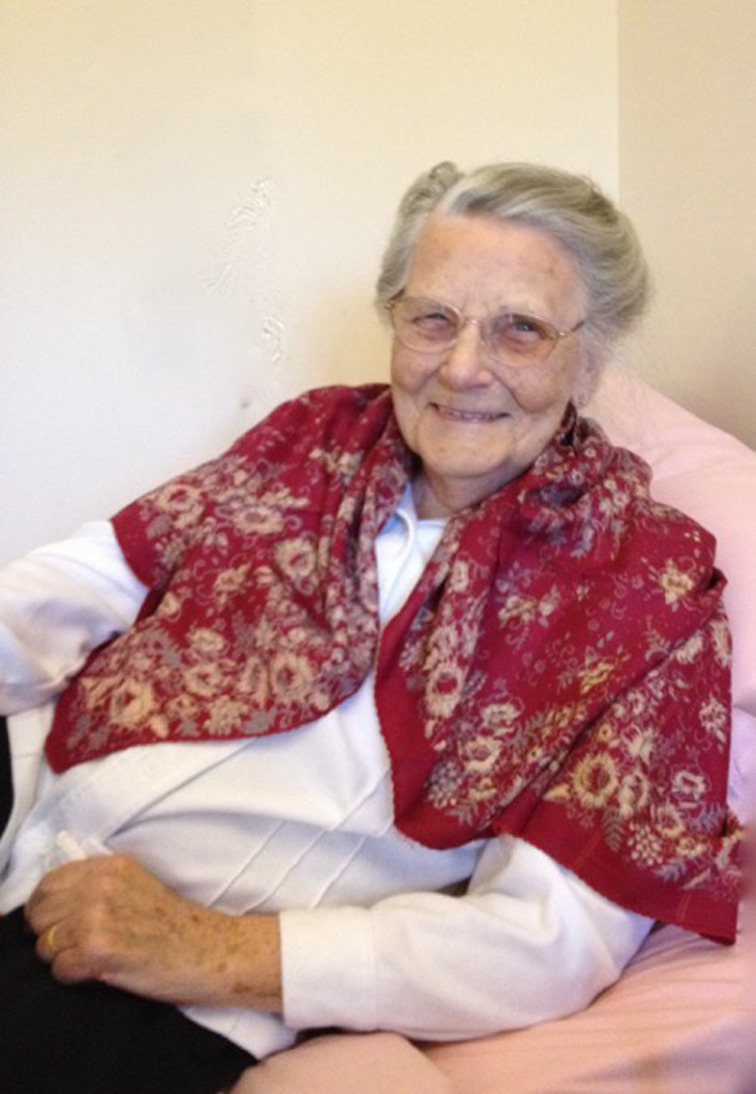

Gwenth (Gwen) Jean Elizabeth Douglas, child psychiatrist and psychoanalyst, who died aged 100 on 22 June 2021, was a pioneer in the treatment of puerperal psychosis. Tom Main, with whom Gwen trained at the Cassel Hospital in London, had previously shown that it was possible to admit seriously ill non-psychotic mothers with their babies to an in-patient unit. Gwen, however, was the first to describe such an approach with psychotic women who, after childbirth, were suffering from schizophrenia or affective disorders. In 1956, having qualified as a psychoanalyst, she published a short but highly influential paper in *The Lancet* on giving psychotherapy to six mothers with puerperal psychosis who had been admitted with their babies to the Neurosis Unit at the West Middlesex Hospital. She concluded that such mothers could be safely looked after with their babies and successfully treated with psychotherapy either alone, or combined with or following physical interventions.^1^ She was supported by Main, who wrote of ‘the twin dangers of separating mother and child, first and more obvious, to the child and second and as fateful to the mother's confidence in her future capacity as a mother.’^2^ Over subsequent years, treatment on a mother and baby unit gradually came to be accepted as the most effective way to manage post-natal psychotic disorders.

Gwen was born on 4 October 1920 in Papatoetoe (Papatoitoi) on the North Island of New Zealand, the daughter of William (Bill), later Sir William, Jordan, a senior Labour politician and later New Zealand High Commissioner to the UK, and Winifred (née Bycroft). She came with her family to England at 15 and did her medical training at St Andrews University, qualifying in 1944. After qualification she served with the Medical Branch of the Royal New Zealand Air Force. Following the war she married Bill Douglas, a meteorologist, and they moved to Malta, returning to England in 1949 when Gwen trained in psychiatry. She and Bill had a son, Martin.

In 1963 Gwen was appointed to a consultant child psychiatrist post at Sutton Child Guidance Clinic, where she remained till 1986. She later worked as a psychiatrist (together with Betty Tylden and Egle Laufer) in the Obstetric Department of University College Hospital with Professors Nixon and Brandt, investigating the causes of emotional disorders of the puerperium and patients with psychosomatic disorders.

Before retiring as a child psychiatrist, she joined the University College Hospital Psychotherapy Department, and supervised medical students on the student psychotherapy scheme as well as trainee psychiatrists. She retired from the National Health Service in 1991 and continued to practise privately as a psychoanalyst until the age of 86. She was much loved by colleagues, students and patients, who appreciated her warm, undogmatic and imaginative approach to psychoanalysis, which was influenced by her teachers Charles Rycroft and Donald Winnicott. She spoke with a gentle and confident voice without any trace of her New Zealand origins.

In retirement Gwen was a keen flower painter and gardener, who enjoyed receiving friends in her beautiful garden in Banstead. She was an avid reader and loved going to all the latest art exhibitions in London. After 2007 she spent her final years comfortably and happily at the Mary Feilding Guild in Highgate and more recently at Henford House in Warminster. She was predeceased by her husband, who died in 1987, and her son.
